# CD146-positive adipose-derived stem cells subpopulation enriched by albumin magnetic sphere ameliorates knee osteoarthritis pain and promotes cartilage repair

**DOI:** 10.1186/s13018-023-04434-9

**Published:** 2023-12-15

**Authors:** Lianghao Wu, Xu Zhang, Chengqing Yi, Hanru Ren

**Affiliations:** Department of Orthopedics, Shanghai Pudong Hospital, Fudan University Pudong Medical Center, 2800 Gongwei Road, Pudong, Shanghai, 201399 China

**Keywords:** Knee osteoarthritis, Adipose-derived stem cells, CD146, Magnetic sphere, Cartilage

## Abstract

**Background:**

The use of adipose stem cell (ADSCs) subpopulations in cartilage repair remains poorly characterized. In this study, we constructed an albumin magnetic sphere with specific targeting of CD146 (CD146-AMs) for sorting a subpopulation of CD146-positive ADSCs (CD146 + ADSCs) and explored the role of CD146 + ADSCs on joint pain and cartilage repair in rats with knee osteoarthritis (KOA).

**Methods:**

CD146-AMs were prepared and analyzed in materialistic characterization tests. Subpopulations of CD146 + ADSCs were sorted using CD146-AMs. Surface labeling, viability, and proliferation of a subpopulation of CD146 + ADSCs were evaluated in vitro. Molecular characterization of mRNA and protein expression profiles was analyzed by microarray. A rat KOA pain model was established by the iodoacetic acid method, and KOA pain and the promotion of cartilage repair were assessed after treatment with bilateral joint cavity injections of CD146 + ADSCs.

**Results:**

The CD146-AMs prepared in this study had an average particle size of 242.63 ± 6.74 nm, an average potential of 33.82 ± 3.53 mv, and high CD146 targeting and low cytotoxicity. The positive rate of enriched CD146 + ADSCs was 98.21% and showed a high level of stem cell marker expression and good cell viability. Gene and protein expression profiles showed that CD146 + ADSCs have different cellular functions, especially in regulating inflammation. In the KOA model, low, medium and high concentrations of CD146 + ADSCs were able to improve KOA pain and promote cartilage repair in a concentration-dependent trend.

**Conclusions:**

The CD146-AMs prepared in this study were able to safely and efficiently sort out the CD146 + ADSCs subpopulation. The subpopulation of CD146 + ADSCs has a unique molecular profile that ameliorates KOA pain and repairs cartilage damage in rats, providing a new idea for KOA treatment.

## Introduction

Knee osteoarthritis (KOA) is the most common chronic degenerative osteometabolic disease in clinical practice and one of the most common geriatric diseases, associated with aging, trauma, and other factors, and is the leading cause of disability in adults, with a disability rate as high as 53% [1, 2]. The incidence of KOA is increasing year by year, and patients with KOA account for about 15% of the global population, posing a serious threat to human health [3, 4]. KOA is one of the most common chronic degenerative refractory diseases in the clinic, which is typically characterized by cartilage degeneration, including a series of degenerative lesions such as cartilage degradation, subchondral osteosclerosis, and bone marrow lesions, which ultimately cause functional dysfunction such as joint pain and stiffness [5, 6]. Therefore, cartilage degeneration caused by abnormal cartilage metabolism is the main pathomechanism of KOA, and cartilage repair is the main goal of early and mid-term treatment of KOA [7]. Clinically available means of KOA treatment are limited and generally have poor efficacy or high toxicity and side effects, which cannot meet clinical needs [8, 9]. Stem cells have been investigated as an alternative cell source for cartilage regeneration due to their high cell viability and multilineage differentiation capacity [10].

Cartilage defects usually lead to post-traumatic inflammation and are a major challenge for cartilage repair [11, 12]. Studies have shown that mesenchymal stem cells (MSCs) can promote tissue repair by regulating inflammation [13]. MSCs have multidirectional differentiation potential and can differentiate into chondrocytes for cartilage repair under specific conditions, opening up a new avenue for local treatment of KOA [14]. However, the clinical application of MSCs still faces many problems such as the genetic stability of stem cells and hypertrophic chondrogenesis and graft ossification, which prevent them from being effectively used in the clinic [15, 16]. Adipose-derived stem cells (ADSCs) have been shown to be capable of directed differentiation toward adipocytes, chondrocytes and osteoblasts, among others. Due to their advantages of abundant source, easy accessibility and rapid growth, they have great potential for application in several clinical departments [17, 18]. ADSCs act as seed cells with chondrogenic potential, self-renewal ability, and immunomodulatory capacity, making them an ideal tool for KOA therapy that can repair damaged cartilage [19, 20]. However, MSCs are a heterogeneous population that cannot be recognized and separated by a single surface marker, and therefore, the results of clinical trials based on MSCs vary [21, 22]. Therefore, the cellular heterogeneity of MSCs affects the therapeutic efficacy.

CD146 is a single-chain membrane penetrating glycoprotein, a member of the immunoglobulin superfamily with homology to many cell adhesion molecules [23]. CD146 is recognized as a marker for pericytes, which have been identified as the natural ancestors of mesenchymal stem cells [24–26]. In this study, an albumin magnetic sphere with CD146-specific targeting (CD146-AMs) was developed to sort ADSCs, and cell surface markers and biological behaviors were evaluated in vitro. By observing different concentrations of CD146 + ADSCs intervening in the KOA pain model of KOA rats, we investigated their effects on improving the pain threshold of KOA and on cartilage damage repairs in rats, which will provide new ideas for the clinical treatment of KOA.

## Methods

### Materials

Human umbilical vein endothelial cells (HUVEC) and ADSCs were purchased from Sayer Biotechnology Co. Thirty male SPF-grade SD rats were purchased from Shanghai Slaughter Laboratory Animal Co. Fetal bovine serum, culture medium, and 0.25% trypsin were purchased from Gibco. Iodoacetic acid was purchased from SIGMA-ALDRICH. Chitosan cetyl quaternary salt (HQCMC) was purchased from Hubei Hanwei Chemical Co. Fe_3_O_4_ was purchased from Hangzhou Jikang New Material Co. Trizol and BrdU kits were purchased from Shanghai Biyuntian Biotechnology Co. The mirVana™ miRNA Isolation Kit was purchased from Thermo Fisher Scientific. 1,2-dioleoyl-sn-glycero-3-phosphocholine (DOPC) and distearoyl phosphoethanolamine-PEG (DSPE-PEG) were purchased from Averito (Shanghai) Pharmaceutical Technology Co. Dimethyloctadecyl [3-(trimethoxysilyl)propyl] ammonium chloride (GHDC) was purchased from Shanghai Shiyang Chemical Co. Albumin was purchased from Merck KGaA. Cholesterol, dichloromethane, N-hydroxysuccinimide (NHS) and other commonly used reagents were purchased from Sinopharm Chemical Reagent Co. 1-(3-Dimethylaminopropyl)-3-ethylcarbodiimide hydrochloride (EDC) was purchased from Shanghai Hualan Chemical Technology Co. CD146, IL-6, CD105, CD166, CD73, CD90, CD45, CD34 and HLA-DR antibody were purchased from Abcam. Safranine O were purchased from shanghai beinuo Biotechnology Co. YLS-3E electronic analgesic instrument was purchased from Huaibei Zhenghua Biological Instrument Co. The plantar thermal radiation pain meter was purchased from Ugo Basile, Italy, and the automatic multifunction enzyme labeling instrument was purchased from BioTek, USA. CytoFLEX flow cytometer was purchased from Beckman Coulter, USA.

### Preparation of CD146-AMs

DOPC (5 mg), HQCMC (5 mg), DSPE-PEG (2 mg), cholesterol (5 mg), albumin (2 mg), and Fe_3_O_4_ (10 mg) were co-dissolved in 2 mL of dichloromethane, and 6 mL of PBS was added, and the mixture was sonicated and shaken using a probe-based ultrasonicator for 6 min, and the dichloromethane was removed using a rotary evaporator to obtain albumin magnetic spheres (AMs). 1 mg of GHDC was dissolved in 1 mL of isopropanol and mixed with 0.1 mg of CD146 antibody, and the GHDC solution coupled with CD146 antibody (CD146-GHDC) was obtained after standing overnight at 4 °C. CD146-AMs were prepared by mixing 1 mL of AMs and 1 mL of CD146-GHDC, adding NHS and EDC, and stirring for 24 h (h) at 4 °C.

### Characterization test

The particle size and potential of CD146-AMs were tested by a particle size analyzer. The magnetization curves of CD146-AMs were tested by a vibrating sample magnetometer (VSM). The morphology and distribution of CD146-AMs were observed by atomic force microscopy (AFM) and transmission electron microscopy (TEM). The infrared spectra of CD146-AMs were tested by infrared spectroscopy.

### Target recognition properties and in vitro cytotoxicity assays of CD146-AMs

ADSCs and HUVEC cells were prepared into single-cell suspensions, incubated with CD146-AMs for 15 min, Prussian blue staining solution was added to the cell solution, and cell crawls were prepared and then observed under the microscope. In addition, cells captured by CD146-AMs were visualized by scanning electron microscopy (SEM). Twenty microliters of DAPI, 20 μL of CD146-AMs-FITC, and 20 μL of Dil were added to ADSCs cells, and the culture dish was fixed on a fluorescence microscope, and photographed and recorded by immunofluorescence microscopy at different time points, respectively. ADSCs cells were added into 96-well plates for culture, about 1000 cells per well. 3, 6, 9 μM AMs and CD146-AMs were added, respectively, and co-incubated for 15 min. Twenty microliters of MTT was added to each well, and 200 μL DMSO was added after incubation for 2 h. Optical density (OD) values were detected by using an enzyme marker with a test wavelength of 560 nm. Five consecutive days of testing was performed, and the OD values were recorded and the cell growth curves were plotted. ADSCs cells were counted and added into 24-well plates with 4 × 10^4^ cells per well. Nine micromolar of AMs and CD146-AMs was added and co-cultured for 72 h. The cells were operated according to the BrdU kit instructions. BrdU primary antibody and primary antibody were added for incubation, respectively, and finally, DAPI was added for incubation, which was observed and photographed under the microscope.

### Growth and identification of CD146 + ADSCs

ADSCs cells were diluted into a cell suspension of 1 × 10^7^/mL, added to a 2-mL sterile EP tube with 10 μL of CD146-AMs cleaned with DMEM, incubated for 30 min and then placed in a magnetic separation frame for 10 min, and CD146 + ADSCs could be isolated by adding 1 mL of DMEM medium for cleaning. It was incubated at 37 °C in a 5% CO_2_ incubator. The CD146 + ADSCs were cultured to 80–90% fusion for passaging culture, and the 3rd passage (P3), 5th passage (P5) and 7th passage (P7) CD146 + ADSCs were observed and detected, respectively, the cell shape and status were observed by microscopy and hematoxylin–eosin (HE) staining, and the growth curves were plotted after cell counting (Trypan blue staining). Calculation formula for a 16 little grid × 25 little grid blood cell counting plate: Number of cells/mL = (Number of cells in 100 little grid/100) × 400 × 10^4^ × dilution multiple. The surface antigen expression of CD146 + ADSCs, including CD146, CD105, CD166, CD73, CD90, CD45, CD34 and HLA-DR, was detected by flow cytometry.

### Microarray analyses

Total RNA from cells was extracted using Trizol reagent and purified by mirVana™ miRNA Isolation Kit. The concentration, purity, and integrity of total RNA were assessed using a Bioanalyzer 2100 (Agilent). Comprehensive differential expression analysis and functional analysis of ADSCs and CD146 + ADSCs were performed using Agilent human mRNA Array. Raw data were obtained by reading microarrays using Agilent Feature Extraction software. Quantile normalization and data processing were performed on the raw data using GeneSpring GX v13 software (Agilent Technologies). The |log2 Fold change|> 1, *P* < 0.05 was set to screen the differentially expressed genes between groups. Hierarchical clustering was performed using R script. Tree visualization was performed by using Java Treeview (Stanford University School of Medicine, Stanford, CA, USA). Gene ontology (GO) pathway enrichment analysis was performed by WEGO (http://wego.genomics.org.cn/cgi-bin/wego/index.pl).

### Animal modeling

Thirty SD rats were randomly divided into a blank group, a KOA modeling group, a CD146 + ADSCs low-concentration treatment group (1 × 10^6^/mL), a CD146 + ADSCs medium-concentration treatment group (1 × 10^7^/mL), and a CD146 + ADSCs high-concentration treatment group (1 × 10^8^/mL), with six rats in each group. Rats in each group underwent KOA modeling by injecting 50 μL of 25 mg/mL iodoacetic acid into each bilateral knee joint cavity, and rats in the blank group were injected 50 μL of 25 mg/mL saline into each bilateral knee joint cavity. Rats in each treatment group were injected with 50 μL of low, medium, and high concentrations of CD146 + ADSCs in the knee joint cavity of the hind limbs bilaterally. Equal amounts of saline were injected into the knee joint cavity of rats in the KOA model group and blank group. Injections were performed once a week for a total of 4 time.

### Pressure pain test

The pain threshold of rats was determined by an electronic pressure pain meter. The rats were loaded into the fixation bucket, so that the animals were in a comfortable and fixed state, and the flat head was used to apply pressure to the dorsum of the hind feet of both sides of the rats, and the pressure value displayed when the animals chirped or struggled due to pain was the pain threshold value of this animal. The basal pain threshold was measured before the start of the experiment, and the pain threshold was measured at weeks 2–4 after modeling.

### Heat pain test

The thermal pain threshold of the rat's bilateral hind toes was detected by the plantar thermal pain sensitivity tester. The rats were placed in a transparent Plexiglas box and the room temperature was maintained at (25 ± 2) °C. After the rats were quiet, the “ten” mark on the tester was placed in the center of the left hind metatarsal plantar foot and avoided the foot pad. Subsequently, the instrument was turned on, and the time from the beginning to the time when the rats raised their legs to avoid it was taken as the thermal pain threshold of the rats. Each foot was irradiated three times with an interval of 5–6 min. to prevent the rats from being scalded by the thermal radiation, we set the upper limit of time for the thermal pain threshold measurement at 20 s and the upper limit of temperature at 35 °C. The rats were then exposed to the thermal radiation three times with a 5–6 min interval. The experiments were measured 6 h after each pressure pain experiment.

### Histologic examination of knee cartilage and evaluation of the degree of KOA

Four weeks after modeling, after the last pain threshold test, the rats were killed by neck-breaking and the knee joints of the five groups were fixed in 4% paraformaldehyde for 48 h, decalcified in EDTA for 4 weeks, dehydrated in alcohol step by step, and embedded in immersion wax. The subchondral cancellous bone of the lateral tibial plateau and the subchondral cancellous bone of the lateral femoral condyle were sliced in the direction of the longitudinal axis of the lower limb on a slicer in the sagittal plane of the knee joint, with a slice thickness of 5 μm, and the slices were routinely deparaffinized in water. For histological examination, the samples were stained with immunohistochemistry staining of IL-6 (Abcam, 1:100) and safranine O. The Mankin’s score was performed with reference to Mankin′s soft group histology scoring criteria (Table 1) [27].

**Table 1:** Mankin’s scoring criteria

Classification	Scoring
*Structure*	
Normal	0
Uneven surface	1
Uneven surface with pannus	2
Cracks to transition layer	3
Cracks to radiation layer	4
Cracks to calcified layer	5
Disrupt completely	6
*Cell*	
Normal	0
Slight decrease, cellular disorders	1
Moderately decrease, clustered distribution	2
Severe decrease	3
*Double staining*	
Normal	0
Slight decrease	1
Moderately decrease	2
Severe decrease	3
No staining	4
*Tide line integrity*	
Intact	0
Crossing with blood vessels	1

### Statistical methods

Data were processed using SPSS22.0 statistical software, and the measurement information was expressed as mean ± standard error, and comparisons were analyzed by one-way ANOVA. Comparison of the two groups was performed by LSD- *t*-test, and *P* < 0.05 was taken as statistically significant difference. A difference of < 0.05 (*P < 0.05; **P < 0.01; ***P < 0.001) was considered statistically significant.

## Results

### Material preparation and experimental procedures

**Fig. 1:**
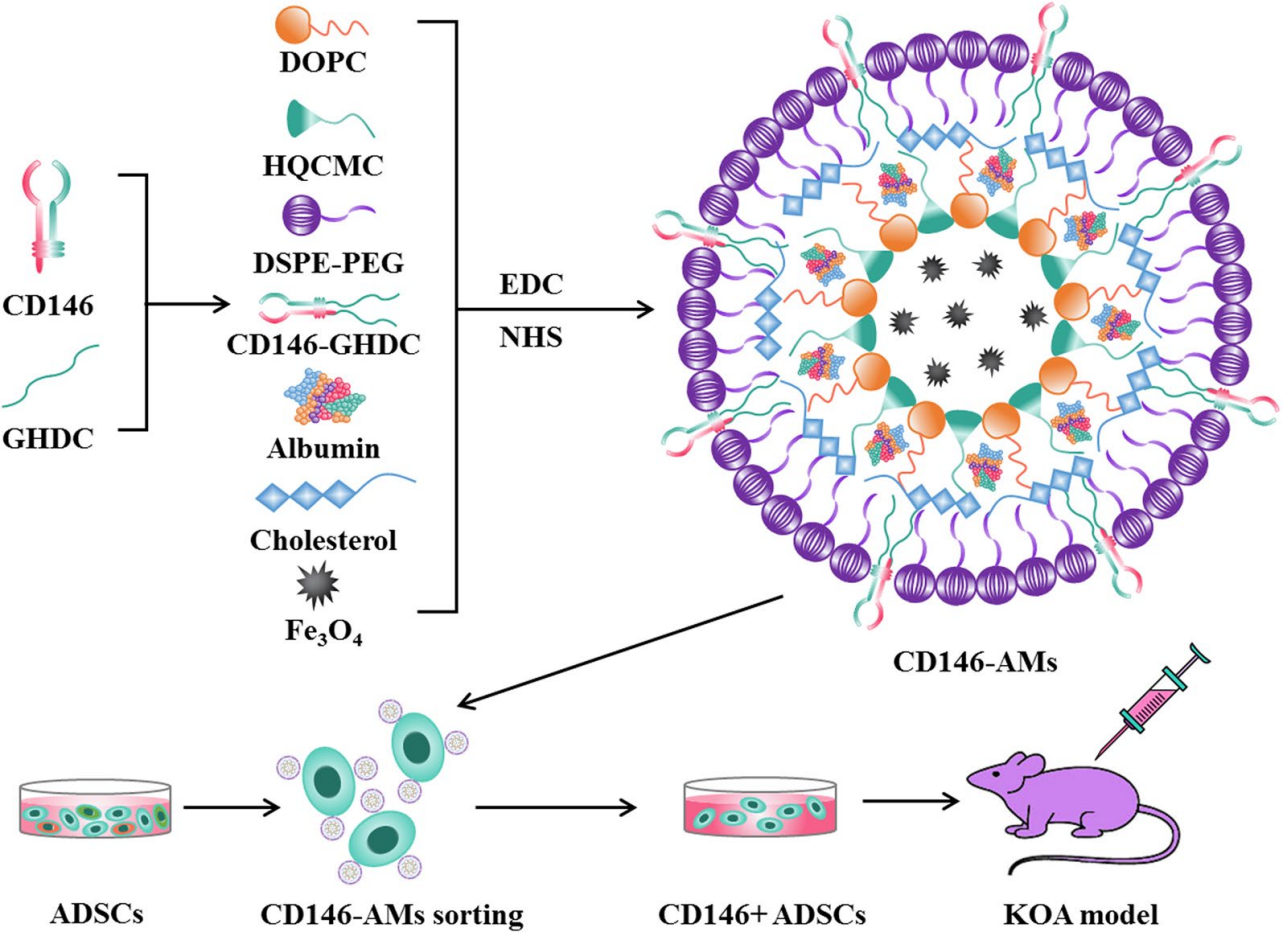
Preparation of CD146-AMs and experimental flowchart

In this study, CD146-AMs were prepared by rotary evaporation. AMs were prepared by co-solubilizing DOPC and other materials in dichloromethane, and the addition of albumin could improve cell targeting and biosafety. CD146-AMs were prepared by adding CD146-GHDC to AMs solution under the effect of coupling agents NHS and EDC. CD146-AMs were added to ADSCs, and CD146 + ADSCs were sorted out for amplification and culture and were injected into the knee joint cavity of rats to improve the pain of KOA and to promote cartilage repair (Fig. 1).

### Characterization analysis

**Fig. 2:**
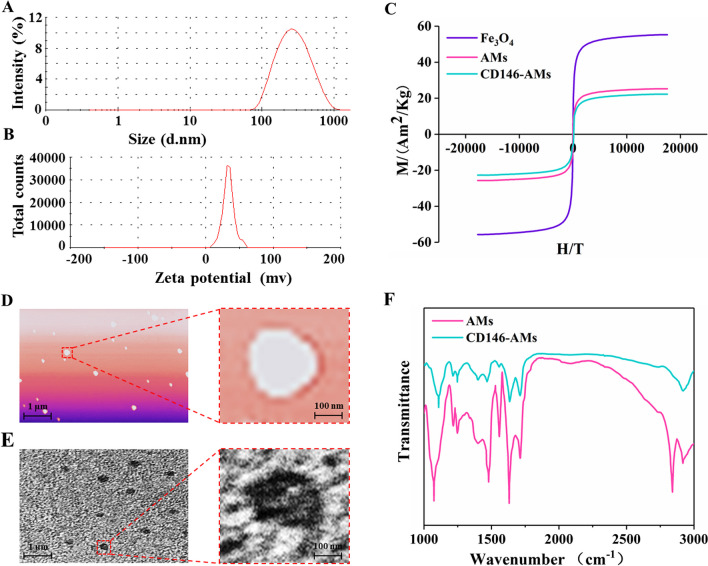
Characterization analysis. **A** Particle size test of CD146-AMs; **B** potential test of CD146-AMs; **C** magnetization curve test; **D** AFM observation graph of CD146-AMs; **E** TEM observation graph of CD146-AMs; **F** infrared test

The average particle size of CD146-AMs was 242.63 ± 6.74 nm, and the polydispersity index (PDI) was 0.187 (Fig. 2A). The average potential of CD146-AMs was 33.82 ± 3.53 mv (Fig. 2B). The magnetization curves showed a saturation magnetization intensity of 55.72 Am^2^/Kg for Fe_3_O_4_ and a decrease in the saturation magnetization intensity of 25.68 Am^2^/Kg and 22.51 Am^2^/Kg for AMs and CD146-AMs, respectively (Fig. 2C). CD146-AMs were observed by AFM and TEM to be granular with a size distribution around 200 nm (Fig. 2D, E). CD146-AMs showed a new infrared peak at 2800–2900 cm^−1^, which was the infrared absorption peak of hypomethyl group, indicating the presence of GHDC on CD146-AMs, and GHDC was coupled with CD146 antibody, suggesting that CD146 antibody had been coupled on the surface of CD146-AMs (Fig. 2F).

### Target recognition properties and in vitro cytotoxicity assays of CD146-AMs

**Fig. 3:**
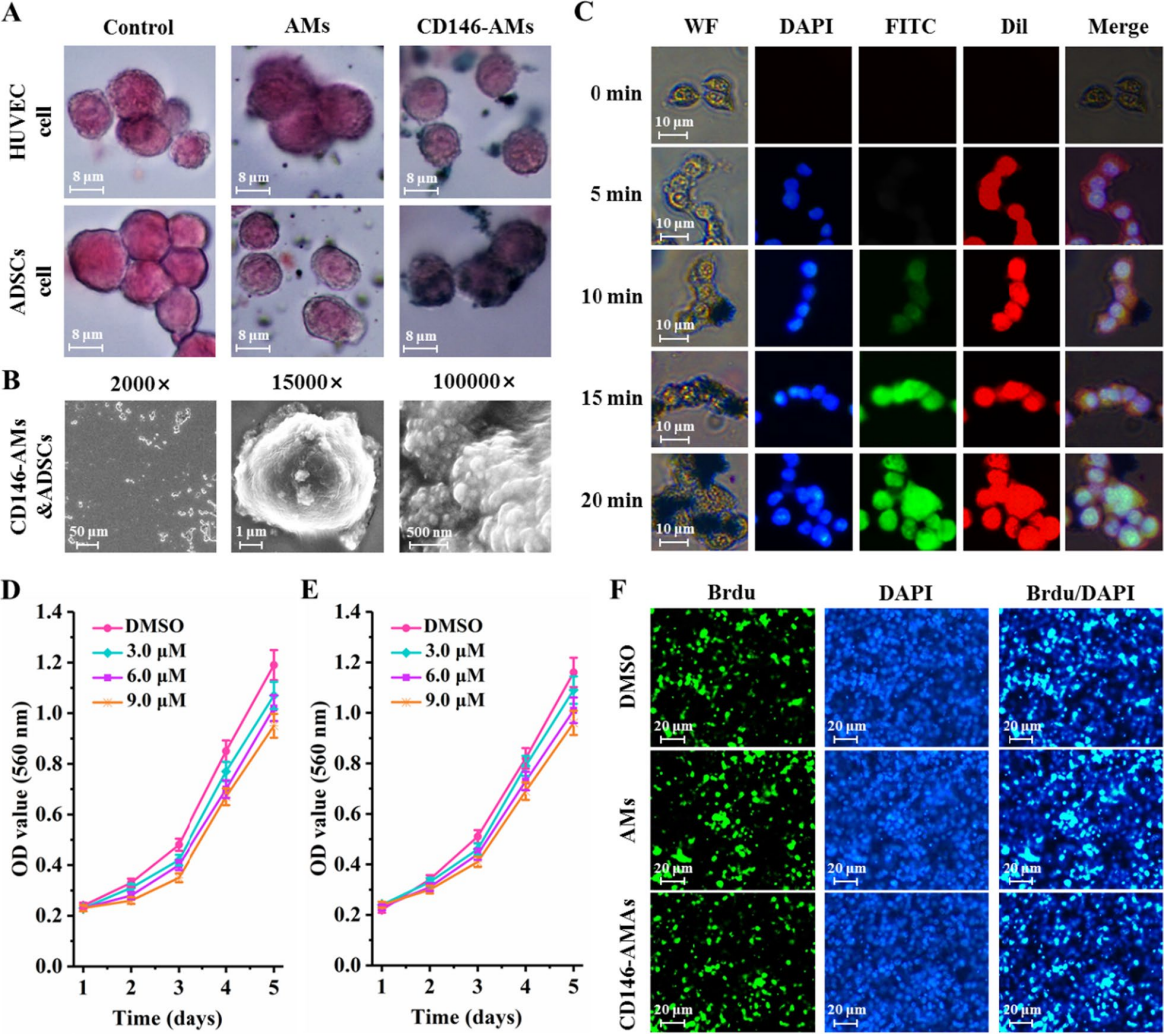
Target recognition properties and in vitro cytotoxicity assay of CD146-AMs. **A** Prussian blue staining of CD146-AMs after binding to cells; **B** SEM observation of CD146-AMs after binding to ADSCs cells; **C** exploration of the binding time of CD146-AMs to ADSCs; **D** effects of adding different concentrations of CD146-AMs on the growth of HUVEC cells; **E** effects of adding different concentrations of CD146-AMs on the growth of ADSCs cells; **F** BrdU staining

Prussian staining results showed that AMs and CD146-AMs were freely distributed around HUVEC cells, AMs were freely distributed around ADSCs cells, and CD146-AMs were mainly adhered to the cell surface of ADSCs cells, suggesting that CD146-AMs were able to recognize ADSCs cells in a targeted manner (Fig. 3A). SEM observation showed that a large number of CD146-AMs were adsorbed and aggregated on the surface of ADSCs cells, and the aggregation of these CD146-AMs on the cell surface facilitated cell sorting (Fig. 3B). The fluorescence staining results showed that the fluorescence signal of FITC in ADSCs cells was gradually enhanced with time, indicating that more and more CD146-AMs were adsorbed on the cell surface, and the best effect could be achieved at 15 min of incubation (Fig. 3C). The addition of different concentrations of CD146-AMs had less effect on the growth of HUVEC and ADSCs cells, indicating that CD146-AMs had less cytotoxicity (Fig. 3D). BrdU staining results showed that there was no significant difference in BrdU-positive cell rate between AMs and CD146-AMs groups compared with DMSO-treated group, indicating lower cytotoxicity (Fig. 3F).

### Growth and identification of CD146 + ADSCs cells

**Fig. 4:**
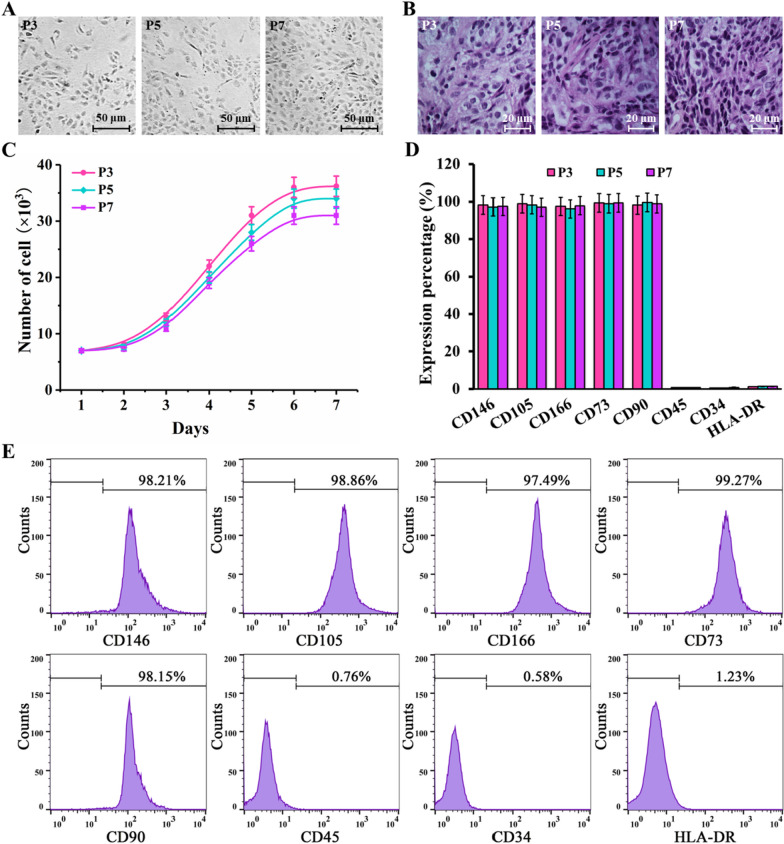
Growth of CD146 + ADSCs cells with surface antigen detection. **A** Growth morphology of CD146 + ADSCs cells; **B** morphology of CD146 + ADSCs cells after HE staining; **C** growth curve of CD146 + ADSCs cells; **D** surface antigen expression rate of CD146 + ADSCs cells; **E** surface antigen expression rate of P3 generation CD146 + ADSCs cells

CD146 + ADSCs cells grew adherently to the wall, and there was no difference in the shape of P3, P5 and P7 generation cells (Fig. 4A). Combined with HE staining showed that the cytoplasm of CD146 + ADSCs cells was mauve in color and the cells grew in aggregates (Fig. 4B). P3, P5 and P7 generation CD146 + ADSCs cells were in growth latency in the first 2 days and in logarithmic growth phase from day 3 to day 6 (Fig. 4C). P3, P5 and P7 generation CD146 + ADSCs cells all highly expressed CD146, CD105, CD166, CD73 and CD90, and CD34, CD45 and HLA-DR were all lowly expressed (Fig. 4D). Among them, P3 generation CD146 + ADSCs cells expressed 98.21% of CD146, 98.86%, 97.49%, 99.27% and 98.15% of CD105, CD166, CD73 and CD90, respectively, and 0.76%, 0.58% and 1.23% (Fig. 4E).

### The subpopulation of CD146 + ADSCs has a unique molecular profile

**Fig. 5:**
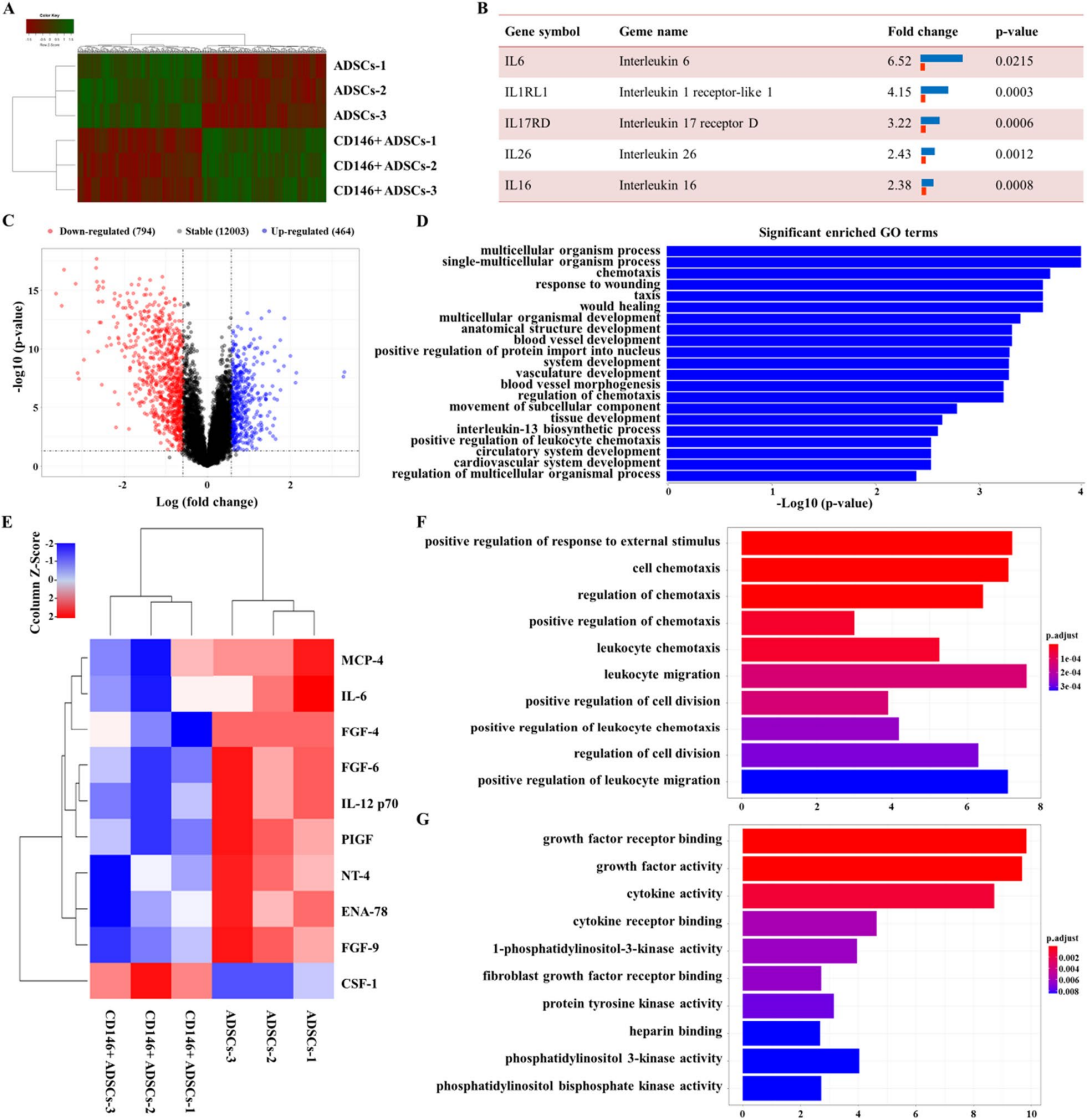
Gene and protein expression in ADSCs and CD146 + ADSCs. **A** Heatmap of differential gene expression in ADSCs and CD146 + ADSCs; **B** inflammation-related genes that underwent significant changes in CD146 + ADSCs cells; **C**. volcano map showing the distribution of gene expression; **D** GO analysis of genes by biological process; **E** heatmap of cytokine expression in ADSCs and CD146 + ADSCs cell subpopulations; **F** GO analysis of proteins by molecular function; **G** KEGG pathway enrichment analysis of proteins

To compare the molecular characteristics of the ADSCs and CD146 + ADSCs subpopulations, mRNA and protein expression profiles of ADSCs and CD146 + ADSCs cells were analyzed by microarray. Differential gene expression was analyzed by clustering biological replicates in each group (Fig. 5A). A total of 1258 differentially expressed genes were identified, of which 794 genes were up-regulated and 464 genes were down-regulated in CD146 + ADSCs cells (Fig. 5C). The expression of immune- and inflammation-related genes, such as IL6, IL1RL1, IL17RD, IL26 and IL18, was significantly increased in CD146 + ADSCs cells (Fig. 5B). GO categorization of genes by biological processes showed that differentially expressed genes were involved in a variety of cellular biological processes such as chemotaxis, response to wounding, taxis, would healing (Fig. 5D). Protein expression profiles of ADSCs and a subpopulation of CD146 + ADSCs were examined by antibody microarrays, and 10 differentially expressed proteins were identified, including immune and inflammatory proteins such as IL6 and IL12 (Fig. 5E). GO and KEGG pathway enrichment analysis showed that the differentially expressed proteins play a role in cell chemotaxis and cell division (Fig. 5F, G).

### The CD146 + ADSCs subpopulation improves KOA pain and promotes cartilage repair

**Fig. 6:**
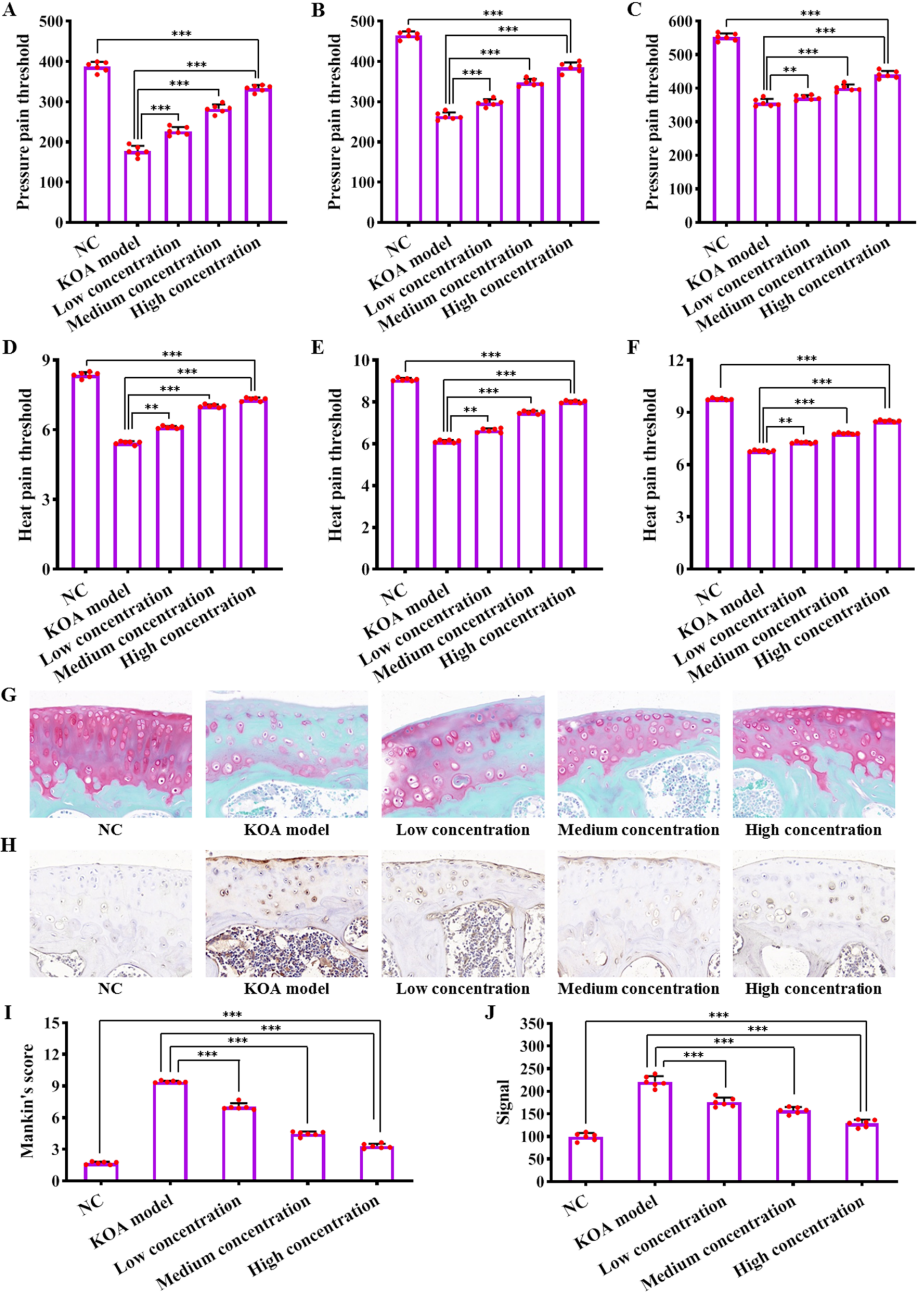
Pressure pain threshold, thermal pain threshold and histopathological detection of knee cartilage in rats. **A** Pressure pain threshold measurement in rats at week 2 after modeling; **B** pressure pain threshold determination in rats at week 3 after modeling; **C** determination of pressure pain threshold in rats at week 4 after modeling; **D** determination of thermal pain threshold in rats at week 2 after modeling; **E** determination of thermal pain threshold in rats at week 3 after modeling; **F** determination of thermal pain threshold in rats at week 4 after modeling; **G** Safranine O staining of rat knee joint cartilage tissue (50×); **H** immunohistochemical staining of IL-6 (40×); **I** Mankin’s score for histopathology of rat knee joint cartilage; **J** analysis of IL-6 expression in cartilage tissues of rat knee joints

The results of pressure pain experiments showed that the pressure pain threshold in the KOA model group was significantly lower than that in the blank group at weeks 2, 3 and 4 after modeling, and with the increase in the therapeutic concentration of CD146 + ADSCs cells, the pressure pain threshold was significantly higher and showed a concentration-dependent trend (Fig. 6A–C *P *< 0.05). The results of thermal pain experiments showed that the thermal pain thresholds of the KOA model group were significantly lower than those of the blank group at weeks 2, 3 and 4 after modeling, and the pressure pain thresholds were significantly higher with the increase of the therapeutic concentration of CD146 + ADSCs cells, with a concentration-dependent trend (Fig. 6D–F *P *< 0.05). Pathological results showed that the cartilage surface of the knee joint of rats in the KOA model group was obviously defective, with severe loss of chondrocytes at the defect, degradation of proteoglycan, and fibrotic degeneration of the subchondral bone. The low concentration group still showed cartilage surface defects, chondrocyte loss and hypertrophic phenotype. In the medium concentration group, a large number of chondrocytes survived, but there were still surface defects and hypertrophic degeneration. In the high concentration group, cartilage was basically restored to normal with thickened cartilage surface and only a few hypertrophied chondrocytes. It showed that low, medium and high concentrations of CD146 + ADSCs had an improvement effect on rat knee joints, with a concentration-dependent trend (Fig. 6G). Compared with the KOA model group, the Mankin′s scores of the CD146 + ADSCs low concentration group, the CD146 + ADSCs medium concentration group and the CD146 + ADSCs high concentration group gradually decreased, and the Mankin′s gradually approached the blank control group as the concentration of the CD146 + ADSCs cell therapy increased (Fig. 6I *P *< 0.05). Meanwhile, the expression of inflammatory factor IL-6 was assessed by histologic methods. IL-6 was highly expressed in the KOA model group, and IL-6 expression in cartilage tissues of the CD146 + ADSCs low, medium, and high concentration groups gradually decreased in a concentration-dependent trend. Therefore, CD146 + ADSCs can reduce inflammation and promote cartilage repair in rat knee joint tissues (Fig. 6H, J, *P *< 0.05).

## Discussion

Commonly used drugs for clinical treatment of KOA include steroidal anti-inflammatory drugs, non-steroidal anti-inflammatory drugs, opioid analgesics, and joint nutrients, which can relieve pain and other symptoms of KOA and improve joint mobility [28]. However, these drugs not only fail to reverse the pathologic process of KOA, but also have significant toxic side effects on the organism [29]. ADSCs are derived from adipose tissue, easy to culture and proliferate, and can differentiate into chondrocytes to promote cartilage repair and effectively inhibit the progression of osteoarthritis (OA). Compared with bone marrow stem cells, ADSCs also have the ability to differentiate into osteoblasts and chondrocytes, and they have the advantages of less trauma, no immune rejection, convenient and repeated sampling, and wide sources, so ADSCs have certain advantages over bone marrow stem cells in the treatment of OA [30–32]. However, the heterogeneity of MSCs affects the cellular therapeutic effect, which hinders their application in regenerative medicine [21, 22, 33]. It has been found that some subpopulations have stronger cartilage formation ability, and the application of subpopulations is becoming a new idea for cartilage repair [34, 35].

CD146 plays a key role in cell adhesion, embryonic development, immune response, angiogenesis and cancer as a surface marker expressed by pericytes and vascular smooth muscle cells [36, 37]. Studies have shown that CD146 defines functional subpopulations of progenitor cell populations and exhibits higher developmental potential [26, 38–40]. However, the role of CD146-positive cells as seed cell types in cartilage tissue engineering is not fully understood. In this study, we prepared a type of CD146-AMs with high CD146 targeting and low cytotoxicity, and successfully sorted out a subpopulation of ADSCs cells with a CD146-positive rate of 98.21%, and the expression of MSCs-associated surface markers remained almost unchanged after sorting. In addition, our microarray analysis data emphasized the multiple functions of the CD146 + ADSCs cell subpopulation in chemotaxis, wound healing, and inflammatory processes, confirming that the CD146 + ADSCs cell subpopulation is functionally distinct from ADSCs.

In this study, the arthropathological examination of the rat knee joint cavity after injection of iodoacetic acid showed gross articular surface, cell necrosis and synovial hyperplasia, and inflammatory cell infiltration, as well as progressive aggravation of joint destruction with the prolongation of time, which was similar to the manifestation of OA, and at the same time, iodoacetic acid-induced knee joints of the rats had a good homogeneity and comparability [41, 42]. The iodoacetate model has certain advantages in OA pain compared with the anterior cruciate ligament transection model, and there are also studies that replicate OA animal models by iodoacetate and suggest that iodoacetate is associated with OA pain [43, 44]. The results of the pain assay in this study showed that CD146 + ADSCs cells could promote cartilage repair and effectively increase the pain threshold of KOA in rats. To a certain extent, high concentration of CD146 + ADSCs cells had a better effect on cartilage repair and improvement of pain. The results of arthropathology showed that the joint cavity injection of CD146 + ADSCs could effectively alleviate the pain of KOA and promote the effect of cartilage repair. These results all suggest that specific subtypes of ADSCs cells will be a new direction for tissue engineering research. The present study is still in the experimental stage, and the mechanism of pain relief and the repair process of damaged articular cartilage by CD146 + ADSCs are still unclear, and a lot of basic research and clinical experience are still needed before conclusions can be drawn.

## Conclusion

The CD146-AMs prepared in this study have high CD146 targeting and low cytotoxicity and can efficiently sort out the CD146 + ADSCs subpopulation. The CD146 + ADSCs subpopulation has unique molecular features that can effectively relieve KOA pain and promote cartilage repair. This study highlights the important role of CD146 + ADSCs subpopulation-based MSCs therapy in tissue engineering, suggesting that the study of functional characterization of specific subtypes of MSCs is a new direction for tissue engineering research.

## Data Availability

The datasets used and/or analyzed during the current study are available from the corresponding author on reasonable request.

## Abbreviations

ADSCs: Adipose stem cell
CD146 + ADSCs: CD146-positive ADSCs
OA: Osteoarthritis
KOA: Knee osteoarthritis
AMs: Albumin magnetic spheres
CD146-AMs: Albumin magnetic spheres with CD146-specific targeting
MSCs: Mesenchymal stem cells
HUVEC: Human umbilical vein endothelial cells
HQCMC: Chitosan cetyl quaternary salt
DOPC: 1,2-Dioleoyl-sn-glycero-3-phosphocholine
DSPE-PEG: Distearoyl phosphoethanolamine-PEG
GHDC: Dimethyloctadecyl [3-(trimethoxysilyl)propyl] ammonium chloride
NHS: *N*-Hydroxysuccinimide
EDC: 1-(3-Dimethylaminopropyl)-3-ethylcarbodiimide hydrochloride
AFM: Atomic force microscopy
TEM: Transmission electron microscopy
SEM: Scanning electron microscopy
OD: Optical density
